# Secular trends in age at menarche and time to establish regular menstrual cycling in Japanese women born between 1930 and 1985

**DOI:** 10.1186/1472-6874-12-19

**Published:** 2012-07-16

**Authors:** Michie Hosokawa, Setsuko Imazeki, Hideki Mizunuma, Toshiro Kubota, Kunihiko Hayashi

**Affiliations:** 1 Faculty of Health Care, Takasaki University of Health and Welfare, Takasaki City, Gunma Prefecture, Japan; 2 School of Health Sciences, Gunma University, Maebashi City, Gunma Prefecture, Japan; 3 Department of Obstetrics and Gynecology, Hirosaki University School of Medicine, Hirosaki City, Aomori Prefecture, Japan; 4 Department of Comprehensive Reproductive Medicine, Tokyo Medical and Dental University, Bunkyo Ward, Tokyo, Japan

## Abstract

**Background:**

Early life-stage exposure to estrogen increases the risk of breast cancer. The objective of this study was to investigate the age at menarche and time to onset of regular menstrual cycles for Japanese women born between 1930 and 1985.

**Methods:**

A cross-sectional study was designed using data from the baseline survey of the Japan Nurses’ Health Study. The data from 48,104 female nurses were analyzed. To view trends in age at menarche, the distribution of age at menarche was calculated for each birth year cohort. The distribution of time to onset of regular menstrual cycles was calculated for each birth year cohort. To estimate whether high-risk group of the estrogenic dependent disorders increase with succeeding generations, we defined the women who experienced menarche at ten years old or younger and started a regular cycle within one year as early age onset of ovulatory cycles.

**Results:**

Average ages at menarche were as follows: 13.8 years for those born in the 1930s (n = 113), 13.3 years for the 1940s (n = 4,751), 12.8 years for the 1950s (n = 15,844), 12.3 years for the 1960s (n = 20,547), 12.2 years for the 1970s (n = 6,568), and 12.2 years for the 1980s (n = 281). The proportion of women who experienced the onset of regular menstrual cycles 1 year after menarche was 29.3% for those born in the 1930s, but decreased to 11.9% for the 1980s. On the other hand, the proportion of women who did not have regular menstrual cycles was 10.4% for those born in the 1930s, but rose to 19.8% in 1980s. The proportion of women who experienced menarche at 10 years old and started regular menstrual cycles within one year increased over time: the percentage was 0.0%, 0.4%, 0.6%, 1.1%, 1.3%, and 2.1% for the women born in 1930s, 1940s, 1950s, 1960s, 1970s, and 1980s, respectively.

**Conclusions:**

The age at menarche of Japanese women born between 1930 and 1985 decreased, but the onset of regular menstrual cycling is delayed; so that the distribution of the start time of ovulatory cycles may have spread for younger generations. Those suggest that the high-risk group of estrogenic dependent diseases among Japanese women may increase in the near future.

## Background

Age at menarche is decreasing worldwide as well as in Japan [1-5]. Puberty is the period during which secondary sexual characteristics begin to develop and the capability of sexual reproduction is attained. Anovulatory cycles are frequently observed in the period immediately after menarche, with menstrual cycles being irregular in many cases. In the later stages of puberty, however, activation of a positive feedback system in the central nervous system leads to the onset of normal menstrual function. Irregular menstrual cycles have a considerable effect on women’s health [6], such as psychosocial stress [6], dysmenorrheal, infertility [7] and further cardiovascular disease in later life [8]. Therefore, from the viewpoint of public hygiene, attention must be paid to when regular menstrual cycles start. But thus far, little is known on the onset of regular menstrual cycles in general population of Japanese women.

It is well known that earlier onset of menarche and regular menstrual cycle is a risk factor of estrogen dependent diseases such as breast cancer [9]. The incidence of breast cancer among Japanese women has been increasing in recent years [10], and early age at menarche is considered as one possible associated factor. Accordingly, it is important to clarify tendencies in age at menarche and time to onset of regular menstrual cycles for different age brackets. The objective of this study is to survey trends concerning age at menarche and time to onset of regular menstrual cycles in Japanese women.

## Methods

### Materials and methods

This study used some of the data from the baseline study for the Japan Nurses’ Health Study. This is a prospective occupational cohort study of female nurses, undertaken with the objectives of investigating the effects of lifestyle and healthcare practices on the health of Japanese women. We announced recruitment to the study at conferences of the Japanese Nursing Association and the Japan Menopause Society, by advertisements in newsletters sent to members of the Japanese Nursing Associations, by invitation from the Japan Nurses’ Health Study Recruitment Committee. Interested medical institutes or individual nurses requested baseline questionnaire sets from the JNHS coordination center by application postcard, facsimile, e-mail, or telephone. At some nurses’ conferences and hospitals, we distributed the baseline questionnaire sets directly to individual nurses. Nurses were informed of the purpose and procedures of the study in the invitation letter, and women who agreed filled out the self-administrated questionnaire with a written consent sheet and replied by mail. The detail of the study design of the Japan Nurses’ Health Study has been reported elsewhere [11].

### Subjects

Subjects were 49,927 female nurses born from 1926 to 1985, and responded to the baseline survey of the Japan Nurses’ Health Study. Their nursing qualifications comprised licenses as registered nurses, licensed practical nurses, public health nurses, and midwives. Women who were born before 1929, who were uncertain year of birth, or who didn’t report age at menarche were excluded from this analysis, and eventually data from 48,104 women born between 1930 and 1985 were analyzed.

### Questionnaire

The Japan Nurses’ Health Study self-administered baseline survey questionnaires were collected by post between 2001 and 2007. Subjects included residents of all 47 Japanese prefectures. Answers to the question about age at menarche were divided into nine categories: ≤9, 10, 11, 12, 13, 14, 15, 16, and ≥17 years old at menarche. The question about time to onset of regular menstrual cycles was phrased as “How many years did it take your menstrual cycle to become regular since your first period (menarche)?” with answers divided into five categories: <1 year, 1–2 years, 3–4 years, ≥5 years, and still irregular. In addition, menstrual regularity at the age of 18–22 years was also asked, with answers divided into five categories: very regular, usually regular, usually irregular, always irregular, or no periods.

### Statistical analysis

Age at menarche was regarded as 9 for those who answered ≤9 and 17 for those who answered ≥17, and average values were calculated. Years of birth were divided into decades, e.g. those born in 1930–1939 were categorized as born in the 1930s. To view trends in age at menarche, the distribution of age at menarche was calculated for each birth year cohort. Changes over time in average age at menarche between birth year cohorts were evaluated using one-way analysis of variance (ANOVA) and Tukey’s multiple comparison test. The distribution of time to onset of regular menstrual cycles was calculated for each birth year cohort. Kruskal-Wallis test was used for the analysis of the differences in the distribution of time to establishment of regular menstrual cycles, and of the differences in the distribution of menstrual regularity at the age of 18–22 years old, in each birth year cohorts. This test was also used for the analysis of the differences in the distribution of the time to onset of regular menstrual cycles and age at menarche. Spearman’s rank correlation coefficient was used to assess their correlation with the time to onset of regular menstrual cycles and age at menarche. Previous study has stated that early menarche and rapid establishment of cycles (within one year) had an almost fourfold increased risk of breast cancer compared with women with later menarche and longer duration of irregular cycles [9] . To estimate whether high-risk group of the estrogenic dependent disorders increase with succeeding generations, we defined the women who experienced menarche at ten years old or younger and started a regular cycle within one year as early age onset of ovulatory cycles. Significance of trends in early age onset of ovulatory cycle’s distribution over time was assessed with the Cochran-Armitage test for trend. P values of ≥0.05 were considered statistically significant. SAS ver.9.2 statistical software was used for data analysis.

### Ethics

The institutional review board of Gunma University and the National Institute of Public Health reviewed and approved the study protocol.

## Results

### Changes over time in age at menarche

**Figure 1:**
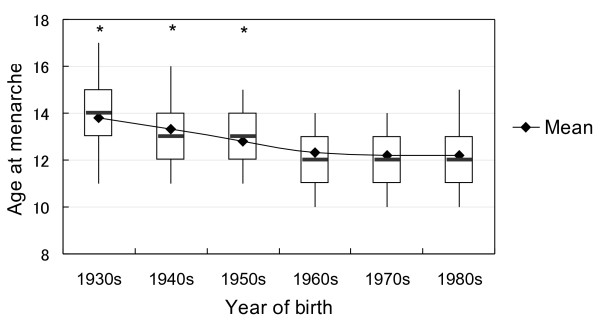
**Box whisker shows the secular changes of mean age at menarche for women born between 1930 and 1985.** Heavy line, Median; box, 25-75%; whiskers, 95% confidence interval. * There was significant difference between mean age at menarche born in the 1980s and those born in the 1930s, 1940s and 1950s.

Age at menarche for each birth year cohort is shown in Figure 1. The overall average age at menarche was 12.6 years (Standard Deviation 1.34), but average ages at menarche for different birth year cohorts (SD) were 13.8 (1.53) for those born in the 1930s (n = 113), 13.3 (1.38) for the 1940s (n = 4,751), 12.8 (1.29) for the 1950s (n = 15,844), 12.3 (1.28) for the 1960s (n = 20,547), 12.2 (1.32) for the 1970s (n = 6,568), and 12.2 (1.39) for the 1980s (n = 281), respectively. The average age at menarche for Japanese women has decreased by 1.6 years for the past 50 years. As shown in the Figure 1, there were significant differences in age at menarche between those born in the 1980s and those born in the 1930s, 1940s, and 1950s, but there was no significant difference between those born in the 1980s and those born in the 1960s or 1970s.

### Interval from menarche to the onset of regular menstrual cycles

**Figure 2:**
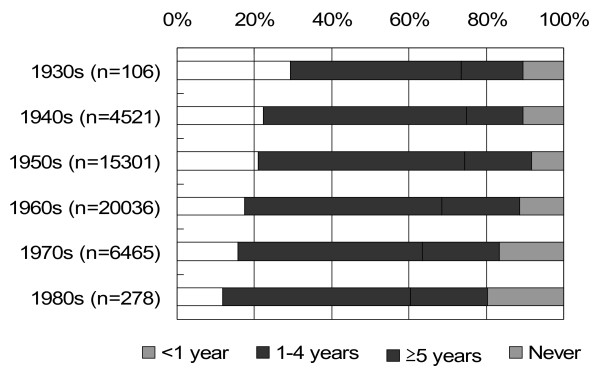
**Distribution of interval from menarche to the onset of regular menstrual cycles by year of birth.** Kruskal-Wallis test χ2 = 428.0, p < .0001.

Interval from menarche to the onset of regular menstrual cycles is shown in Figure 2. The proportion of women who experienced the onset of regular menstrual cycles <1 year after menarche was 29.3% for those born in the 1930s, but decreased to 11.9% for the 1980s. On the other hand, the proportion of women who did not have regular menstrual cycles was 10.4% for those born in the 1930s, but rose to 19.8% in 1980s (Kruskal-Wallis test χ^2^ = 428.0, p < .0001). These data indicate that the interval from menarche to the onset of regular menstrual cycles has lengthened with succeeding generations.

### Menstrual regularity at the age of 18–22 years old

Text for this sub-section Table 1 shows the regularity of menstrual cycles at the age of 18–22 years old by birth decade. The proportion of women who had regular menstruation (very regular + usually regular) during the age of 18–22 years old was significantly higher in older generations (Kruskal-Wallis test χ^2^ = 312.0, p < .0001). This is consistent with the finding that the onset of regular menstrual cycling is delayed in women with later birth decade.

**Table 1:** Distribution of menstrual regularity at age of 18–22 years old by year of birth

	**Year of birth**										
	**1930s**	**1940s**	**1950s**	**1960s**	**1970s**	**1980s**					
	**n (%)**	**n (%)**	**n (%)**	**n (%)**	**n (%)**	**n (%)**					
Menstrual regularity						
Very regular	42 (42)	1664 (38)	5935 (38)	6933 (34)	1965 (30)	55 (20)
Usually regular	32 (32)	1772 (40)	6090 (39)	7855 (39)	2468 (38)	126 (45)
Usually irregular	19 (19)	661 (15)	2365 (15)	3815 (19)	1386 (21)	69 (25)
Always irregular	6 (6)	282 (6)	1013 (7)	1559 (8)	622 (10)	28 (10)
No periods	2 (2)	39 (1)	118 (1)	193 (1)	78 (1)	2 (1)

Kruskal-Wallis test χ2 = 312.0, p < .0001.

When Figure 1 and Figure 2 are viewed together, we can see that the age at menarche of Japanese women has decreased over succeeding generations, but the interval from menarche to the onset of regular menstrual cycling has extended. We therefore analyzed the interval from menarche to the onset of regular menstrual cycling by age at menarche. As shown in Table 2, the proportion of women who experienced the onset of regular menstrual cycles in 5 years and more after the menarche and who did not experienced regular cycles was significantly higher in women whose menarche occurred at 15 years old and over (Kruskal-Wallis test χ^2^ = 75.4, p < .0001), suggesting that later onset of menarche is accompanied with longer duration of irregular menstrual cycle.

**Table 2:** Distribution of interval from menarche to onset of regular menstrual cycles by age at menarche

	**age at menarche**		
	**≤10**	**11-14**	**≥15**
	**n (%)**	**n (%)**	**n (%)**
time to onset of regular menstrual cycles			
<1 year	439 (18)	7797 (19)	558 (17)
1-2 years	739 (31)	12356 (30)	879 (27)
3-4 years	558 (23)	8875 (22)	640 (19)
≥5 years	414 (17)	7618 (19)	681 (21)
Never	225 (9)	4388 (11)	540 (16)

Kruskal-Wallis test χ2 = 75.4, p < .0001.

**Figure 3:**
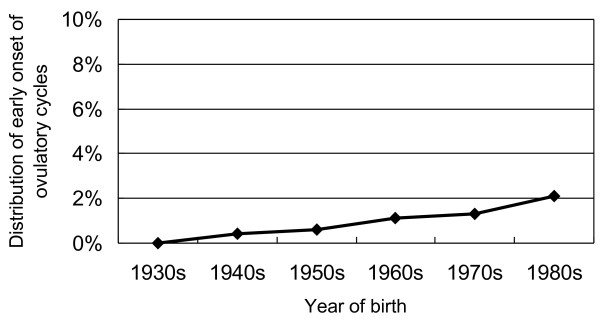
**Distribution of early age onset of ovulatory cycles by year of birth.** Early age onset of ovulatory cycles means the woman who experienced younger age at menarche at ten years old or younger and started a regular cycle within one year. Cochran-Armitage Trend Test Z = -7.40, p < .0001.

In order to investigate whether early age onset of ovulatory cycles is increasing in younger generations, distribution of the woman who experienced menarche at ten years old or younger and started a regular cycle within one year as early onset of ovulatory cycles was studied. As shown in Figure 3, the proportion of early age onset of ovulatory cycles was significantly increasing in younger generations of Japanese women (Cochran-Armitage Trend Test Z = -7.40, p < .0001), 0.0% for those born in the 1930s, 0.4% for those born in the 1940s, 0.6% for those born in the 1950s, 1.1% for those born in the 1960s, 1.3% for those born in the 1970s, 2.1% for those born in the 1980s, respectively.

## Discussion

Age at menarche has decreased worldwide [1-5]. The results of the present study show that the average age at menarche of Japanese has decreased by 1.6 years for past 50-years since 1930. According to previous study, the average age at menarche for Japanese women born between 1881 and 1900 was 15.1 years old, but this had decreased to 12.5 years for those born between 1960 and 1970 [12]. The present study confirmed the trend of decreasing age at menarche in Japanese women reported by previous study [12], and moreover covered the results of women born after 1970 until 1985. Although our results showed significant decrease in age at menarche by 1.6 years during the 50-year period from 1930 to 1980, the speed of the decrease of age at menarche was decelerated with younger generations. As shown in Figure 1, age at menarche significantly declined among subjects born between 1930’s and 1950’s, but the decline was not significant anymore in those born in 1960’s and thereafter, suggesting that age at menarche of Japanese women reaches nadir. Thus, the results of the present study are compatible with the findings by Rees [13], who reported that although age at menarche has been decreasing in developed nations for the past 100 years, this tendency has slowed and might even be reversing.

Various factors have been suggested to be associated with age at menarche, including body mass index (BMI), race, size at birth, genetic factors, intrauterine conditions, nutrition, other stresses, light-darkness cycle and climatic conditions, exposure to endocrine-disrupting chemicals [14-17].

By birth cohort, average birth weights were increasing as follows: 1930’s, 3.16 kg; 1940’s, 2.91 kg; 1950’s, 2.95 kg; 1960’s, 3.01 kg; 1970’s, 3.08 kg; 1980’s, 3.08 (p < .0001). Terry showed negative association between birth weight and age at menarche [17], the present study did not obtain any data on BMI at age of menarche but on birth weight. To assess associations between birth weight and age at menarche, we used logistic-regression models for earlier age at menarche relative to later age at menarche. The results showed negative slight association between birth weight and age at menarche (data are not shown). This association is similar to the study by Terry [17]. However, the association between birth characteristics and age at menarche has been less consistent [15]. The association between birth characteristics and age at menarche remains to be investigated. However since we do not have data of other factors, we do not conclude that only birth weight has influenced earlier menarche. Moreover, Yoshinaga [18] has reported that the prevalence of obesity among elementary students is increasing, but Hermanussen’s [19] data show no rise in the BMI of Japanese children, and investigation is also required of the association between BMI of Japanese children and menarche.

As shown in Figure 2, the interval from menarche to obtain regular menstrual cycle has extended with succeeding generations, and the proportion of women who did not have regular menstrual cycles increased. According to previous studies, a common etiology for amenorrhea is weight loss, related to dieting and a desire for thinness. And an increase in athletic activities, emotional stress related menstrual pattern [20]. The increasing of the proportion of women who has irregular cycles in our study may be affected by the change of the life style with the times such as nutrition. We asked participants time to onset of regular cycles using five categories, we could not calculate the exact age at start of regular menstrual cycles. As shown in Table 1, the percentage of subjects who had regular menstrual cycles at age of 18–22 years old was higher in older generations than younger one. This finding supports a study on French women by Clavel-Capelon et al [21] who showed that the interval from menarche to the onset of regular cycling showed a steady increase, and that the percentage of women in whom regular cycling started at least 5 years after menarche was increased in the younger generations. Interval from menarche to the establishment of regular cycling was very weakly positively correlated with age at menarche in the present study; (Spearman’s r =0.044, p < .0001) and was compatible with the results obtained by the Nurses’ health study in US women (Spearman’s r = 0.09) [22]. These means that in younger generations the average age at menarche of Japanese women decreased, but the onset of regular menstrual cycling may not decreased likewise, so that it can be said that the distribution of the start time of ovulatory cycles may have spread for younger generations. The discrepancy between the earlier onset of menarche and delayed acquirement of regular menstrual cycling is a matter of interest, but thus far we have no data that can physiologically account for. However, we infer that the discrepancy may cause generation-specific diseases, in particular estrogen dependent disorders.

A previous study has shown that a woman with early menarche and immediate establishment of regular cycles were risk factors for breast cancer [9]. From these two risk factors on menstruation, we supposed women who had menarche before ten years old and regular cycling within one year after the menarche were being at high-risk of breast cancer. The distribution of such high-risk group has been significantly increasing among younger generations of Japanese women, suggesting that estrogen dependent diseases may increase in the near future among Japanese women.

There are several limitations in this study. As the present study gathered data on age at menarche recollected by subjects aged 25–70, recall bias must be taken into account. In addition, the question about regularity of menstrual cycles did not define this term, and answers may have been affected by the subject’s judgment. However, Must [23] reported that 407 (91%) of 448 women were able to recall their age at menarche, and that there was a strong correlation with actual values (r = 0.79, p < 0.001). We asked participants time to regular cycles using five categories, we couldn’t calculate age at onset of regular cycles. We defined high risk group by women experienced menarche by 10 years old and regular cycles within one year.

This study covered female nurses, who are likely to take a greater interest in their own health than do women in general, and who have knowledge of health and healthcare as professionals. Therefore, it can be said that their answers concerning age at menarche and regularity of menstrual cycles were highly reliable.

As the present study was a large-scale epidemiological study of women throughout Japan with a large number of respondents, these findings may reflect the state of menarche of Japanese women in general.

## Conclusions

The age at menarche of Japanese women born between 1930’s and 1980’s decreased, and the interval from menarche to the onset of regular menstrual cycling extended successively for the younger generations. Women who underwent delayed menarche experienced delayed menstrual regularity. So that it can be said that the distribution of the start time of ovulatory cycles may have spread for younger generations. The distribution of high-risk group of estrogen dependent diseases has been increasing significantly among younger generations of Japanese women.

## Competing interests

The authors declare that they have no competing interest.

## Authors’ contributions

The following authors were responsible for study concept and design: MH, SI and KH. SI, HM, TK and KH made contributions to acquisition of data. MH performed the statistical analysis and interpretation of data, and drafted the manuscript. HM, TK have been involved in revising it critically for important intellectual content. KH, SI supervised data analysis, and interpretation of data and helped to draft the manuscript. All authors helped review drafts of the manuscript and have read and approved the final manuscript.

## Pre-publication history

The pre-publication history for this paper can be accessed here:

http://www.biomedcentral.com/1472-6874/12/19/prepub
